# Individual differences in behavioural inhibition explain free riding in public good games when punishment is expected but not implemented

**DOI:** 10.1186/1744-9081-9-3

**Published:** 2013-01-10

**Authors:** Anya Skatova, Eamonn Ferguson

**Affiliations:** 1 School of Psychology, University of Nottingham, Nottingham, UK; 2 Horizon Digital Economy Research, University of Nottingham, Nottingham, UK

**Keywords:** Cooperation, Free riding, Punishment risk, Behavioural inhibition, Individual differences

## Abstract

**Background:**

The literature on social dilemmas and punishment focuses on the behaviour of the punisher. However, to fully explain the effect of punishment on cooperation, it is important to understand the psychological mechanisms influencing the behaviour of those who expect to be punished. This paper examines whether the expectation of punishment, rather than the implementation of punishment is sufficient to prevent individuals from free riding. Individual differences in the punishment sensitivity have been linked to both threat responses (flight, fight, fear system, or the FFFS) and to the response to the uncertainty of punishment (BIS-anxiety).The paper, therefore, examines if individual differences in BIS-anxiety and FFFS can explain some of the variability in free riding in the face of implemented and non-implemented punishment.

**Methods:**

Participants took part in a series of one-shot Public Goods Games (PGGs) facing two punishment conditions (implemented and non-implemented) and two standard non-punishment PGGs. The punishment was implemented as a centralized authority punishment (i.e., if one participant contributed less than their group members, they were automatically fined). Individual contribution levels and presence/absence of zero contributions indexed free riding. Individual differences in behavioural inhibition were assessed.

**Results:**

Individuals contributed more under the threat of punishment (both implemented and non-implemented). However, individuals contributed less when the punishment was *not* implemented compared to when it was. Those scoring high in BIS-anxiety contributed more when the punishment expectations were not implemented. This effect was not observed for FFFS.

**Conclusion:**

Supporting previous research, punishment had a powerful effect in increasing contribution levels in the PGGs. However, when expected punishment was not implemented, individual differences in punishment sensitivity, specifically in BIS-anxiety, were related to fewer contributions (increased free riding) as compared to the situation when punishment was not implemented. This has implications for our understanding of why some people cannot resist the temptation to free ride, even when facing possible punishment for their actions. Our findings suggest that the diminished functioning of mechanisms, associated with trait behavioural inhibition, can partly explain such behaviours.

## Background

Cooperation is a fundamental feature of human society. Yet cooperation often has a cost implication and consequently people are not always cooperative, and choose instead to free ride. Free riding is reduced, however, when it is punished [1-4]. Different forms of punishment are effective in eliminating free riding and sustaining cooperation, such as peer or altruistic punishment (when members of the group punish/fine other free riding group members, at their own cost), central authority punishment (if someone contributes less than their group on average they are fined through an automated rule) and third party punishment (free riders are punished by a person who is not a part of their group) [5,6]. Most research has focused on how different forms of punishment affect cooperation levels and when and why individuals choose to punish free riders [3,7]. However, to understand when sanctions effectively sustain cooperation, it is important to understand the psychological mechanisms influencing the behaviour of those who receive or are threatened with punishment: what makes them choose to cooperate?

There are relatively few studies that have examined the psychological mechanisms of why some individuals cooperate in social dilemmas and others do not. Scheres and Sanfey [8] explained cooperation via strategic reciprocation (cooperating when you expect others to cooperate in return) and individuals’ reward sensitivity, see also [9]. The current experiment examined if a different, but related, psychological mechanism, sensitivity to punishment, can explain some of the variability in responses to punishment in the social dilemma scenario [10]. Individual differences in sensitivity to punishment predict greater behaviour responses under conditions of threat and punishment [11,12]. As such, punishment sensitivity may plausibly explain some of the variation in free riding under the threat of punishment, within the context of a Public Goods Game.

### Public goods games and punishment risk

In a typical PGG several participants (usually four) are matched to play together in a group and are given an endowment of Monetary Unites (MUs). They are aware that they can keep these MUs for themselves, or invest the MUs into a public account. All money invested into the public account is multiplied and distributed equally to all group members. If everybody from the group invests equally, then all group members get equal returns and increase their profit. If individuals do not invest equally, those who invested less (the free riders) ultimately receive more money than those who invested more, because everybody gets an equal return from the public good. This scenario creates a dilemma: an opportunity to cooperate or to free ride. One effective strategy to reduce free riding is to introduce a punishment system, whereby free riders can either be punished by other players or by a centralized rule (a central authority (CA) punishment). As a result, Public Goods Games (PGGs) with punishment provide a model of social sanctions against free riding [13].

In laboratory PGG experiments, cooperation increases as a result of punishment for free riding [13,14]. In real life, the exact likelihood of punishment is often unknown and individuals have to estimate the likelihood of punishment based on experience: such as whether an expected punishment occurred or not. For example, there may be fixed speeding limits and fines for exceeding them, however, there may be times when one speeds but no fine is forthcoming: a police officer did not notice the speeding car, the speed camera was not functioning, the speed may was only slightly over the limit. Similarly in a legal system, a crime have varying punishment alternatives (a formal caution or warning, fine or community sentence), or the person may not be caught at all. Indeed evidence suggests that individuals make a judgement regarding behaviour which is partly based on how likely punishment is to occur in a given situation based on their own experience and the prevailing conditions [15].

The majority of the research on punishment in social dilemmas has focused on peer-punishment. It has been demonstrated that uncertainty about whether a punishment will be implemented leads to more cooperation and a reduction in free riding, at least in a peer-punishment scenario [16]. Uncertainty affects punishment levels in the PGGs: for example, even if the information about behaviour of group partners (if they cooperate or free ride) is not reliable [17], people still punish, even though their net payoffs decrease [18]. In a peer-punishment PGG experiment, Fudenberg and Pathak [19] demonstrated that individuals contributed more in an ‘observed’ punishment condition (i.e. after each game participants were informed about the punishment points they received), than in an ‘unobserved’ punishment condition (i.e. all punishment points were revealed at the end of the experiment). The difference in the conditions could be linked to expectations concerning whether or not punishment is going to be implemented. In the observed condition participants received the feedback on how many punishment points did they get after each game, throughout the experiment. In the unobserved condition, punishment was implemented but participants did not have information about their punishment points until the end of the experiment. Therefore, their estimation of the risk of being punished would not have been based on direct observations and their expectation may have been less accurate as a result. In this study we examine how contribution levels change in response to a change in the punishment risk, when the punishment rule is either implemented or not.

A way to examine how the expectation of punishment and the certainty of its administration affect individual behaviour is to control levels of punishment experimentally. In the peer-punishment situation a punisher may decide whether or not to fine a free rider depending on whether they consider the extent of the free riding worthy of punishment. For instance, in Fudenberg and Pathak [19], punishment levels received in the games varied between individuals, depending on the amount of punishment partners decided to administer. As the primary goal of our research is to look into the psychological reasons behind behavioural responses to punishment, we used a CA punishment rule in the PGGs to control the delivery of punishment.

A CA punishment design allows for higher experimental control over punishment expectations, with a fixed rule for the punishment of free riding. In the current study, participants were told that punishment was possible if they transgressed a specified rule. However, in one condition participants were actually punished if they transgressed the rule (implemented punishment), and in the other condition they were not punished even if they transgressed the rule (non-implemented punishment). The central question here was: when people break a rule and are not caught, is the threat that the punishment might be applied at some point still sufficient to promote cooperation, or would the unrealised threat of punishment lead people to start free riding again?

The few studies that have looked into the effects of CA punishment on PGG behaviour did not study the psychological mechanisms that may influence contribution levels in response to punishment. For example, Guillen *et al*. [20] demonstrated that individuals cooperated more with CA sanction mechanisms compared to standard PGGs, but with no explanation as to why. This paper aims to answer this question by using a CA punishment rule to study why people cooperate more when punished for free-riding was implemented or not.

### Behavioural heterogeneity in the PGGs and individual differences in behavioural inhibition

A growing body of research has shown heterogeneity in behavioural strategies in PGGs [21]. A wealth of studies [22-24] have explored mechanisms linked to the behaviour of the punisher, but a gap still remains in the literature with regard to understanding the behaviour of those who are punished or expect to be. Any variability in responses to punishment could potentially be accounted for by psychological mechanisms relating to individual differences in motivation [25]. One way to explore the heterogeneity of behaviour associated with punishment is to examine individual differences with respect to individuals’ sensitivity to punishment, which has been shown to predict responding under threat and punishment conditions, as well as to responding to uncertainty [26-28].

Motivational processes, e.g. linked to motivational traits of avoidance and approach [1], should potentially influence how individuals react to punishment. The behavioural inhibition system (or BIS-reactivity), assessed psychometrically (e.g. the BIS scale from the BIS/BAS questionnaire, [29]) has been shown to explain individual behaviour in situations when there is a threat of punishment [29]. Specifically, the BIS has been implicated in risk assessment [10,30], choices under uncertainty [31] and risky behaviour in general, e.g. different forms of dependencies, psychopathologies [27,32]. It is hypothesised, therefore, that BIS-reactivity can explain some of the variance in individual responses in the PGGs with punishment.

While BIS-reactivity is associated with traits such as constraint, self-control and neuroticism, neither BIS-reactivity nor related traits have been studied in relation to levels of free riding in PGGs where punishment is possible. However, a study by Hirsh and Inzlicht [33] demonstrated that individuals with high levels of neuroticism (which is strongly correlated with BIS-reactivity) produced a larger neural response to uncertainty than to negative information presented without uncertainty in the context of a cognitive task. Therefore, it is not clear whether response to uncertainty in punishment expectations (punishment is expected, but it is either implemented or not) would be associated with individual differences in traits like BIS-reactivity or neuroticism.

Furthermore, it has been suggested that BIS-reactivity is not a unified construct either conceptually [26] or in terms of its psychometric assessment [28], but rather comprises two systems: a pure reaction to punishment or the flight, fight, freeze system (FFFS), and a reaction to probable or expected punishment known as BIS-anxiety [10,28]. This distinction has been demonstrated also at the psychometric level. Specifically, the BIS-scale can be re-scored to asses two respective conceptual systems: BIS-anxiety and FFFS [28]. As BIS-anxiety and FFFS differ in their neural and physiological underpinnings, as well as in their behavioural outcomes, cf [26], they should also have a different effect on behaviour in the PGGs, specifically when comparing situations when punishment either was or was not implemented. BIS-anxiety, should more likely be involved in situations of higher uncertainty (e.g., in the condition when punishment was never implemented). Specifically, higher levels of BIS-anxiety should prevent individuals from free riding when punishment is expected but not implemented. Therefore, those with high BIS-anxiety should choose to cooperate to avoid punishment when punishment is expected but non-implemented. Those with lower BIS-anxiety, should choose to free ride more often, as it might lead to a larger immediate individual rewards, despite the risk of being caught. In contrast, we expected FFFS to explain behaviour only in conditions when punishment was actually implemented and participants experienced punishment.

### Experimental design and predictions

In this study we manipulated the implementation of punishment in a CA punishment design. Such design mimics a situation where a known fine or punishment (e.g., speeding, parking) does not occur for every transgression. We investigated how the implementation of punishment or the mere possibility of expected punishment affects contributions in a PGG and how behaviour under these conditions are influenced by BIS-anxiety and FFFS.

**Figure 1:**
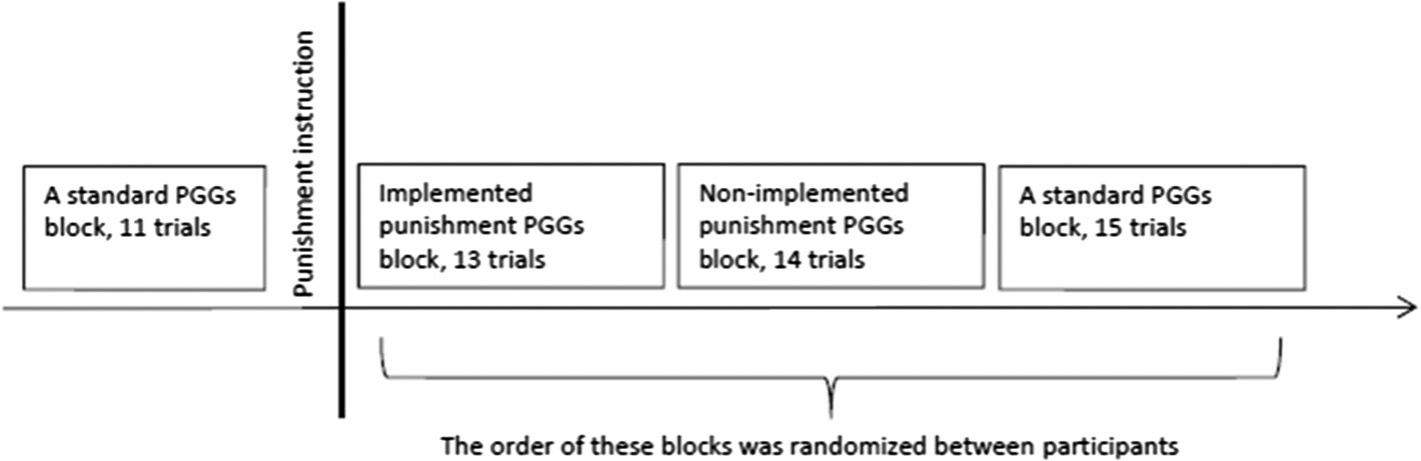
**A schematic representation of the experimental procedure.** See description in the text.

We compared two punishment blocks (labelled as *implemented* and *non*-*implemented*) to two standard PGGs blocks without punishment. To ensure that the punishment instruction did not change the behaviour of participants, the first block was always a standard PGG without punishment. The punishment instruction was then introduced, followed by the remaining three blocks. In addition, to ensure that the order of these three blocks (implemented punishment, non-implemented punishment and a standard game) did not have any effect on the contribution levels, the order of three blocks was randomized (see details below). Figure 1 depicts the schematic representation of the experimental design.

The aim of this study was twofold: (1) to compare contribution levels of PGGs when a risk of punishment was not expected (a baseline standard PGGs block) with the blocks when it was expected, and the expectations were either met (implemented punishment block) or not (non-implemented punishment block), and (2) to investigate if there was an effect of differences in behavioural inhibition on free riding behaviour in implemented versus non-implemented punishment conditions.

In punishment conditions, regardless of whether or not punishment was implemented, lower individual contributions should reflect diminished cooperation and increased free riding. Furthermore, it was predicted that if individuals make choices about contributions taking into account the certainty of punishment, then contributions should be higher when the expectation of punishment was met (i.e. implemented punishment) in comparison to when the punishment was expected but did not occur (i.e. non-implemented punishment). Finally, lower BIS-anxiety should lead to high levels of free riding (lower cooperation) under conditions of non-implemented punishment in comparison to implemented punishment, whilst higher FFFS should lead to increased contributions under implemented conditions.

## Method

### Participants and procedure

The sample consisted of 79 undergraduates (mean age 20.96, ranged from 18 to 36, 67.1% female). Questionnaires were administrated using online software prior to the experiment. The experiment took place in a computer room, where all participants were seated in individual booths with dividers. The experiment was programmed using Z-Tree [34]. Participants received £3 as a show-up fee and they had an opportunity to earn up to £5 extra. On average, participants received £6.67 (equivalent to 10.57 USD) for a one-hour experiment. After finishing the experiment, participants were debriefed and paid individually. The experiment was approved by the School of Psychology, University of Nottingham Ethics Committee. Written consent was obtained from all participants.

### Measures of motivational orientations

Following Heym *et al*. [28], we used four BIS-anxiety items from the seven–item BIS scale from the Carver and White [29] BIS/BAS questionnaire to calculate BIS-anxiety (α = .83). We also calculated the fearfulness (FFFS or flight, fight, fear system) component of BIS-reactivity using the remaining three FFFS items from the same BIS scale (α = .79). We also calculated the overall BIS-reactivity using all seven of Carver and White’s BIS scale (α = .81). There were no predictions with regard to individual differences in BAS; therefore, analysis of this scale was omitted from the results in this paper.

### The public goods game

The experiment comprised four blocks: two standard PGGs (a baseline condition) and two punishment blocks. At the start of the experiment, all participants played one block of standard PGGs. After the first block, participants received additional instructions for the next three blocks, which introduced a punishment rule. The rule stated that in some games they might be “fined” (punished) if they contributed less on average than others in their group (calculated by the computer). They were told that the punishment rule would be applied only in some of the blocks and they were informed before the beginning of each block if the punishment rule operated in that block. Thus if participants were not informed that the punishment rule applied to that block, they knew they were playing a standard PGG. After the punishment instruction was introduced, they played the remaining three blocks of games (two different punishment blocks and a standard PGG without punishment). Participants were not explicitly informed how many blocks they would play.

The two punishment blocks had exactly the same instructions - participants were told that they might receive punishment for free riding. The only difference between punishment blocks was that in the implemented punishment block punishment actually occurred in two of the 13 trials in the block. In the non-implemented punishment block, punishment never occurred. The order of three blocks after the punishment instruction was counterbalanced through six pseudorandom sequences, with approximately the same number of randomly allocated participants (from 12 to 16) in each sequence.

Each block consisted of a number of one-shot games, which are referred to here as trials. Each trial was an anonymous PGG, where participants played in groups of four and had to divide their initial endowment of 20 MUs (equal to £2) into the private and public/group account. After everybody made their decisions, the investments were calculated based on the following pay-off function:

(1) $$\pi_{i}=20-g_{i}+0.5\sum_{j=1}^{4}g_{j}$$

where a pay-off (π) for a participant *i* is defined by their contribution (*g*) and the sum of contributions of other players.

After participants made their decisions and entered their contribution into the group account, they received feedback about how much on average their group contributed and how much they earned. On all trials, the information they received about the group’s average contribution was manipulated. However, in one trial, participants actually played with each other as a group. Accordingly, participants were informed that all of the trials except for one (a real trial) would not be played with people in the room, and for the non-real trials all of the information that appeared on the computer screen would be defined by the experimenter. Participants were made aware that only the earnings from the real trial would count towards their final payment. As a result, no deception was involved in this study, as the trials were organized in a Conditional Information Lottery, see [35], for a discussion. In the debrief participants stated that they were unable to detect which was the real trial and had treated all trials as if they were real.

The feedback about the contribution of other group members for each participant was manipulated through the design. Participants received feedback about a high average group contribution level (between 16 and 18 MUs) in two trials of each block, and in the rest the feedback was medium (between 7 and 12 MUs). Including two high contribution trials in each block ensured that the majority of participants could experience punishment in the implemented punishment block (as participants were only punished if they contributed less than the group average on these high contribution trials), but also that the structural parameters (contribution levels of group partners) of the games did not vary across conditions. A varied schedule of medium and high contribution levels was introduced to imitate natural variability in the behaviour of group partners, thus preventing participants from detecting high contribution trials. Different numbers of trials in each block ensured that participants could not predict how many trials there were in each block (see Figure 1).

To investigate if motivational traits were associated with free riding in the games we used both individual contribution levels and whether or not a participant contributed zero at least once in each condition as behavioural outcomes. Both variables have previously been used to index individual cooperation and free riding in the PGGs, e.g. [36,37].

## Results

### Effects of punishment on contribution levels

**Figure 2:**
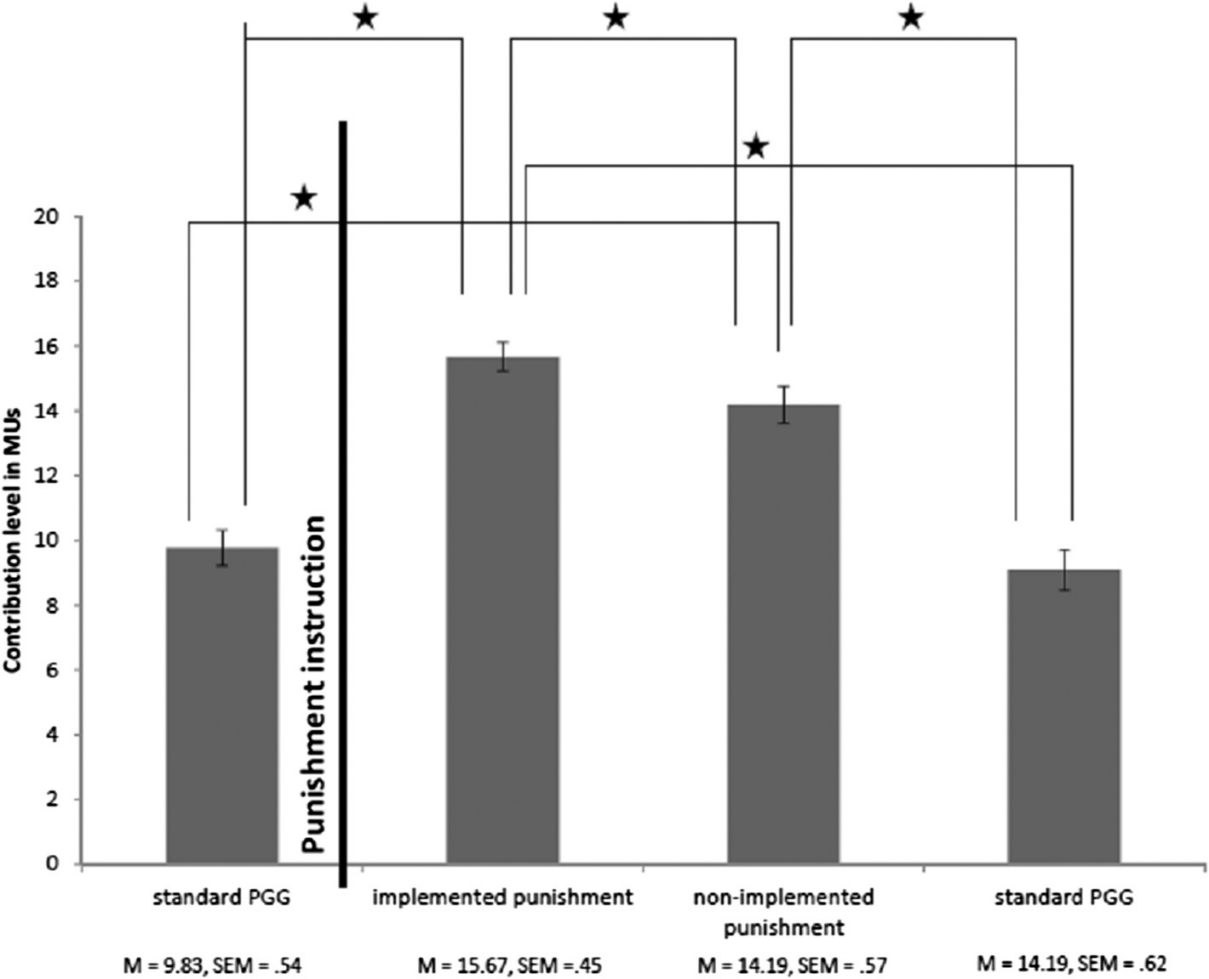
**The mean (M) contribution levels for each block.** Error bars represent standard errors of the mean, SEM). "*" denotes *p* < .001 significance level.

To explore if there was a main effect of different punishment conditions, a two-way mixed ANOVA, on average contribution levels, was conducted. There was a within-subjects factor of block (four levels – four blocks) and a between-subjects factor of sequence (the order of the blocks). The results revealed a main effect of block (*F*_(3,219)_ = 63.61, *p* < .001), no effect of the sequence (*F*_(5,73)_ = 1.71, ns) and no significant interaction of sequence with block (*F*_(15,219)_ = 1.59, ns), demonstrating that the pseudorandom ordering did not affect individual contribution levels in each block. Figure 2 summarizes the results of the main effect of block.

Post-hoc tests were conducted, with a Bonferroni correction, demonstrating that the mean contribution levels of the initial standard PGG block were lower compared to the implemented (*t* (78) = −10.39, *p* < .001) and non-implemented punishment blocks (*t* (78) = −7.15, *p* < .001); the mean contribution level of a standard PGG block after the punishment instructions was lower compared to the implemented (*t* (78) = −10.70, *p* < .001) and non-implemented punishment blocks (*t* (78) = −7.47, *p* < .001). Thus, participants contributed more in blocks when they either experienced punishment^a^ or when there was a possibility of punishment compared to two standard PGGs blocks.

Contributions in the standard PGGs blocks were not significantly different from each other (*t* (78) = 1.40, *p* = .193): participants contributed similarly in the standard PGG block before they received the instruction about possible punishment as compared to the standard PGG block after they received the punishment instruction.

Contributions in the block when the punishment was implemented were significantly higher than when the punishment was not implemented (*t* (78) = 4.11, *p* < .001).

It could be possible that participants who experienced an implemented punishment block prior to the non-implemented punishment block (N = 40) were more likely to expect punishment in the non-implemented punishment block as compared to those who had experienced these blocks in reverse order (N = 39). There were no significant differences in the contribution levels between those who experienced the implemented punishment block before the non-implemented punishment block as compared to those who had experienced these blocks in the reversed order: the implemented punishment block first (*t* (77) = −1.41, ns), the non-implemented punishment block first (*t* (77) = −1.28, ns). Therefore, the order of implemented punishment versus non-implemented punishment blocks had no effect on choices in the PGGs.

### Behavioural inhibition and the difference score

**Figure 3:**
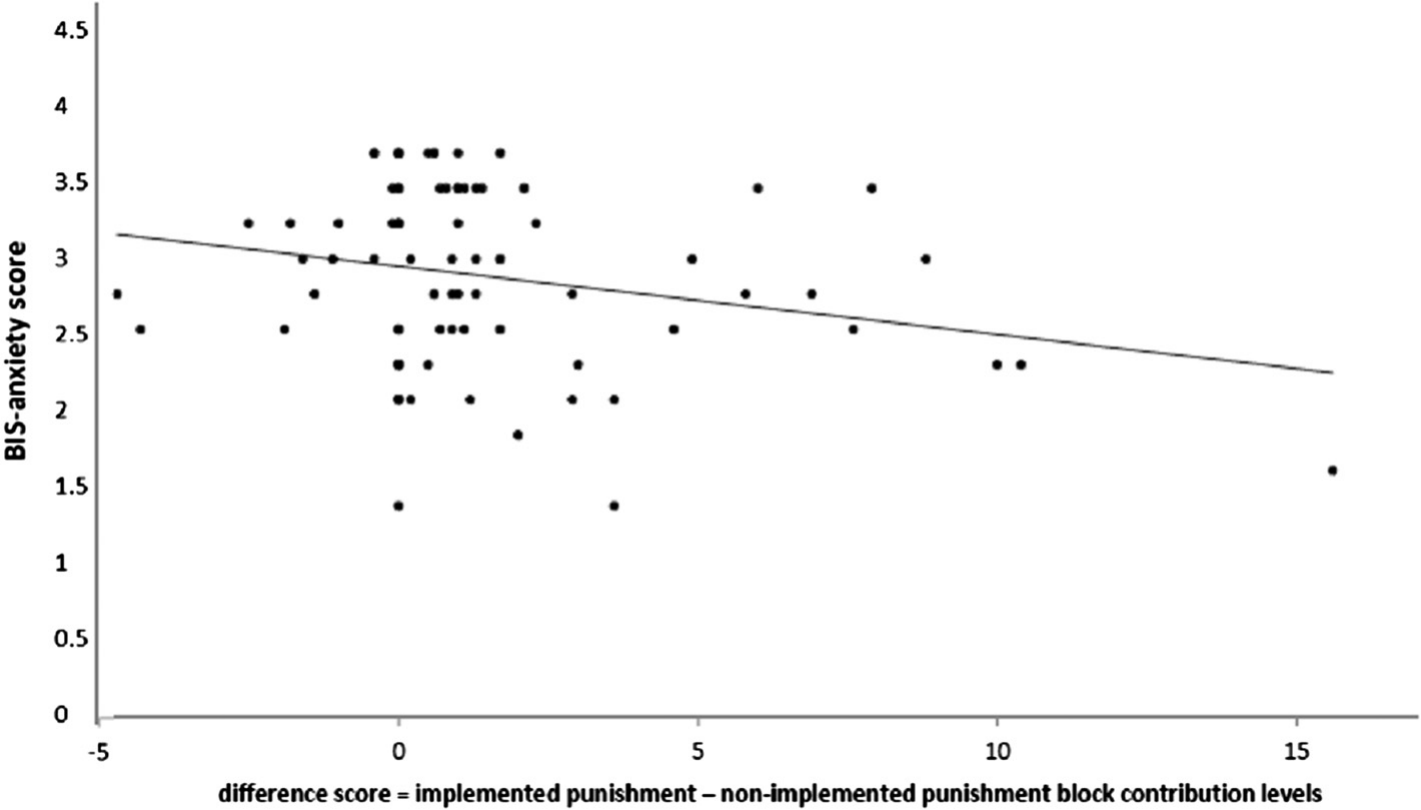
**Association of BIS-anxiety with the difference in contribution levels between the implemented and non-implemented punishment condition.** A positive difference score means that a participant decreased their contribution in a non-implemented condition compared to the implemented condition. The trend line represents linear regression estimation.

The means (M) and standard deviations (SD) for BIS-reactivity were M = 2.93, SD = .56, for BIS-anxiety: M = 3.13, SD = .64, and for FFFS: M = 2.68, SD = .61. It was expected that those higher in BIS-anxiety should contribute more when the punishment is expected but not implemented. A difference score between levels of contributions in the implemented versus non-implemented punishment blocks was calculated (contributions in the implemented punishment condition minus contributions in the non-implemented punishment condition: a positive score indicates that more was given in the implemented condition than the non-implemented condition). This score was used to analyse the effect of behavioural inhibition on changes in contribution levels when punishment was not implemented compared to when it was implemented. This difference score was significantly negatively correlated with BIS-reactivity (r = −.237, *p* < .05) and BIS-anxiety (r = −.240, *p* < .05), but not the FFFS subscale (r = −.173, *p* = .13). Thus while the association of the difference score with both FFFS and BIS-anxiety was negative, the effects for BIS-anxiety were stronger and reached significance level, whereas the association between the difference score and FFFS subscale did not. Figure 3 summarized the results for BIS-anxiety in a scatter plot. It supports the prediction that those high in BIS-anxiety made higher contributions in the non-implemented as opposed to the implemented punishment conditions. Conversely, compared to the implemented punishment condition, those low in BIS-anxiety gave less in the non-implemented punishment condition.

### Behavioural inhibition and zero contributions

**Figure 4:**
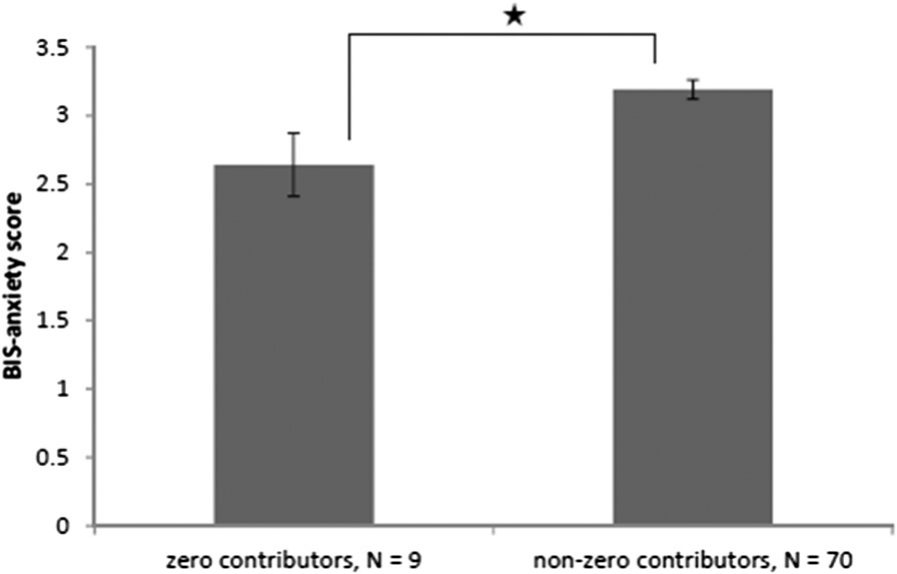
**Differences in mean BIS-anxiety for zero and non-zero contributors, a non-implemented punishment block.** "*" denotes *p* < .05 significance level.

As in Keser and van Winden [37], participants were divided into zero contributors (i.e. those, who made at least one zero contribution across all the games in the block,) and non-zero contributors (i.e. those, who never made a zero contribution during any of the games in a particular block). An independent sample *t*-test demonstrated that in the non-implemented punishment block those who never gave a zero (i.e., always contributed something: N = 70) were significantly higher in self-reported BIS-anxiety (M = 3.00, SD = .61), compared to those who made a zero contribution at least in some of the games (i.e., free rode) (N = 9, M = 2.63, SD = .70; *t* (77) = −2.50, *p* < .05). Figure 4 summarises these findings. The two groups also differed significantly in BIS-reactivity (*t* (77) = −2.80, *p* < .01) (not graphed). Thus in the situation when the punishment was non-implemented those who self-reported higher BIS-anxiety were more likely to contribute at least something compared to those with a lower BIS-anxiety. Due to the small number of participants in one of the groups, we re-ran the analysis (and all subsequent analyses in this section) with a non-parametric Mann–Whitney test. Non-parametric analyses supported the results of all parametric analyses.

Interestingly, relationships between BIS-anxiety and zero contributions approached significance only for one other condition: the standard PGG block after the punishment. On the standard PGG block after the punishment instruction, those who never made a zero contribution (i.e., always contributed) in this block (N = 60) scored higher BIS-anxiety (M = 3.20, SD = .58); compared to those who made zero contribution (i.e., free rode) on at least one of the games in the block (N = 19, BIS-anxiety scores M = 2.89, SD = .78; *t* (77) = −1.83, *p* = .07). This provides additional evidence in favour of the involvement of BIS-anxiety in processing punishment risk contingencies: once the punishment was announced as a condition in some of the games, participants with higher levels of BIS-anxiety avoided contributing nothing (made no zero contributions on any trials of the remaining blocks).

### FFFS and contribution levels under implemented punishment condition

There was no correlation between contribution levels under implemented punishment conditions and FFFS (*r* = −.001, *p* = .97). The relationships between contribution levels in the other blocks and FFFS were also non-significant.

### Behavioural inhibition and free-riding tendencies

To further test the hypothesis that BIS-anxiety should prevent individuals from free riding when facing a possibility of punishment, we examined if BIS-anxiety affects behaviour of those who showed free riding tendencies. The first standard PGGs block, before the punishment instruction, was used to classify participants as free riders and non-free riders. Those, who contributed in this block less than the mean contribution of the group (N = 23), were classified as free riders similar to procedure was used in [38,39]^b^. As predicted, for free riders, BIS-anxiety correlated positively with contribution levels in the non-implemented punishment block (*r* = .433, *p* < .05). Therefore, individuals with free riding tendencies, when punishment not implemented, contributed more only when they were higher in BIS-anxiety.

## Discussion

This study contributes to the literature in several ways. It supports previous findings demonstrating that an expectation of punishment for free riding, whether it is implemented or not, increased individuals’ contributions significantly, compared to conditions without punishment [13,40,41]. The results showed that individuals contributed more when the expectation of punishment was implemented compared to when it was not. Further, the results showed that behavioural inhibition, and specifically BIS-anxiety, prevented individuals from free riding (i.e., led to increased contribution levels) in a situation when punishment was expected but was not implemented. Thus this study shows that the possibility of punishment, whether or not it is implemented, has an influence on the levels of free riding, and subsequently cooperation. However, it further indicates that basic motivational differences in the reaction to punishment and uncertainty (i.e., behavioural inhibition) can explain some of the heterogeneity in these responses.

### Possibility of punishment is enough to increase contribution levels

There have been a number of recent investigations into the effect of punishment on pro-social behaviour in social dilemmas [13,42]. However, these studies mostly employ a “real partners” design, which makes it impossible to experimentally control the feedback about group contribution levels. In those studies, punishment levels depend on the contribution levels of real group partners as well as the behaviour of the “punishers” in response. Thus, it is difficult to assess how likely each individual is to receive a punishment, as this depends not only on their behaviour, but also on the behaviour of their group partners. Thus the advantage of documenting the natural history of the player is gained at the cost of losing some experimental control.

Without deceiving participants [35], the design reported here experimentally controlled the group contribution levels as well as the amount of punishment they received. Punishment contingencies (either implemented or non-implemented) were fixed for all participants and did not depend either on other players’ contribution levels or participants’ individual contribution levels (unless they contributed more than 17 MUs on the “punishing” trial). This design differs from other research on the effects of punishment contingencies on behaviour in the social dilemmas, e.g. [13,42]. Accordingly, the design enabled us to study behaviour in response to the fixed punishment contingencies, revealing the basic regularities of behaviour when facing a punishment or its possibility in the PGGs.

In line with the predictions, the results demonstrated that the contribution levels under the conditions of actual (implemented) punishment were higher than when the punishment was possible but never occurred (non-implemented). This confirms general accounts of broader theoretical models of decision-making, e.g. reinforcement learning models [43], and suggests that the choice of a beneficial decision in each trial was affected by the information about reward/punishment risks. Specifically, in the implemented punishment individuals could estimate the “online” probability of punishment as higher, as they actually experienced the punishment, as compared to non-implemented punishment condition. In cases when the punishment was possible, but never occurred, “online” probability of punishment could be estimated as lower. As a result, they contributed less in the non-implemented punishment condition compared to the implemented.

### Behavioural inhibition affects contribution levels and zero contributions when punishment is expected but not implemented

It was hypothesized that BIS-anxiety would influence behaviours as a function of punishment expectancy. Specifically, when punishment expectancies were not met, as opposed to when they were, individuals high in BIS-anxiety were more likely to contribute compared to those low in BIS-anxiety. The results here supported this prediction. In line with this prediction, those participants, who reported higher BIS-anxiety, were more likely to keep their contributions at a high level even when punishment was expected but not implemented. Furthermore, those who were higher in BIS-anxiety invested something on all trials of the non-implemented punishment. Those who were lower in BIS-anxiety were more likely to have given a zero contribution (i.e., they free rode) on at least one of the games. The same differences in BIS-anxiety were maintained for the standard PGGs block after the punishment instructions (i.e., after the punishment contingencies were introduced in the game). Therefore, higher BIS-anxiety was associated with decreased free riding behaviour when the punishment was more uncertain: expected but not implemented.

Gray and McNaughton [1] have suggested that trait behavioural inhibition (BIS as opposed to FFFS) should modulate behaviours in situations of uncertainty. In this experiment, uncertainty was manipulated by varying punishment probability. Accordingly, those with higher BIS-anxiety continued to respond in accordance with punishment expectations, even when there was no threat of punishment (the second standard PGG block). The evidence reported here supports broader associations between behavioural inhibition and depression [44], which in turn is linked to overvaluation of punishment contingencies [45] and might lead to risk aversion in the situation of punishment uncertainty.

### Low behavioural inhibition in free riders predicts lower contribution levels when the risk of punishment is low

In line with the predictions, for those who demonstrated free riding tendencies during a standard PGGs block, level of BIS-anxiety predicted behaviour in the punishment blocks. Specifically when punishment was implemented there was no significant correlation between BIS-anxiety and contribution levels for either free riders or non-free riders. However, when punishment was not implemented, previous free riders with higher BIS-anxiety contributed more. This supports the findings of Block and Gerety [46], who showed in a social dilemma that individuals who had previously committed crime (offenders), were more sensitive to changes in punishment risk than university students. It is possible that individuals who showed free riding preferences were more sensitive to the change in punishment risk in the non-implemented punishment condition, where the threat of punishment was not confirmed. From our findings, previous free riders reduced their contribution if they were also lower in BIS-anxiety. Therefore, low BIS-anxiety could be argued to be associated with more accurate assessment of prevailing punishment contingencies, while high BIS-anxiety is associated with overestimation of punishment contingencies.

### Role of BIS-anxiety in threat expectation

Our findings are consistent with broader literature on anxiety and avoidance motivation. As in Hirsh and Inzlicht [33], we found that BIS-anxiety was specifically associated with behavioural change when the certainty of punishment changes, but not with behaviour under the conditions of punishment per se. Specifically, BIS-anxiety did not correlate with individual average contribution levels in each of the two punishment conditions but only with the difference in contributions between conditions. This suggests that BIS-anxiety influences choices in the specific situation when punishment was expected but not implemented. All our effects were significant only for BIS-anxiety. Further, contrary to our predictions, we did not find a correlation between FFFS and contribution levels under implemented punishment. This has implications for our understanding of BIS-anxiety and associated traits (e.g., neuroticism), suggesting that manipulations of uncertainty (for example, through information about behaviour of others or through the probability of punishment for free riding) can have as large effect on individuals higher in BIS-anxiety and their subsequent behaviour, as actual experience of punishment. Further, behavioural outcomes under uncertain punishment conditions will differ as a function of BIS-anxiety. Those who are low in BIS-anxiety should adjust much faster to a decreased probability of punishment, and quicker in switching back to behavioural strategies that were previously punished.

## Conclusion

Conforming to the body of previous research, e.g., [39,42,47], punishment for free riding has a profound effect on contribution levels in the PGGs: when the punishment is expected, whether it is implemented or not, aggregate cooperation increases. However, when punishment is expected but not implemented some individuals choose to free ride, while others continue to cooperate. We showed that individual differences in BIS-anxiety can help to differentiate levels of free riding when punishment becomes less certain (expected but not implemented). This finding may help explain why some people cannot resist the temptation to free ride, even when facing a possible punishment for their actions. Our findings suggest that reduced functioning of mechanisms associated with trait behavioural inhibition are related to improved punishment risk assessment, which in turn can explain such behaviours.

## Endnotes

^a^ Not all participants received a punishment in the implemented punishment block, as the rule for being punished was contributing less than group partners, which was 16 or 17 MUs. 74.7% participants experienced punishment. The rest never experienced punishment, as they contributed more than group average on the “punishing” trials. Additional analyses with only contribution levels of participants who were punished did not change significance of any results.

^b^ By the structure of the game, mean average contribution was 7 MUs. Therefore, under the conditions of this experiment individuals free rode only when contributed less than 7 MUs on average. Instead, those, who contributed more than 7 MUs did not free ride (on average).

## Competing interests

The authors declare that they have no competing interests.

## Authors’ contributions

AS participated in designing the study, carried it out, analysed the data and drafted the manuscript. EF participated in designing the study, drafting and commenting on drafts of the manuscript and analyses. Both authors read and approved final manuscript.
